# A Methodology for In-Well Multiphase Flow Measurement with Strategically Positioned Local and/or Distributed Acoustic Sensors

**DOI:** 10.3390/s23135969

**Published:** 2023-06-27

**Authors:** Ömer Haldun Ünalmis

**Affiliations:** Weatherford, Houston, TX 77041, USA; haldun.unalmis@weatherford.com

**Keywords:** fiber-optic measurements, downhole flow measurement, multiphase flow, sound speed measurement, distributed acoustic sensing (DAS)

## Abstract

A new three-phase downhole flow measurement methodology is developed based on measurements of speed of sound at different locations along the well, where the pressure is greater than the bubble-point pressure at the first location and smaller at the second location. A bulk velocity measurement is also required at the second location. The fluid at the first location is a mixture of two phases, but becomes a mixture of three phases at the second location due to the liberation of gas from the oil phase. The flow equations are first solved for two-phase flow at the first location to obtain the first phase fraction, water-in-liquid ratio, and then this information is fed into the flow equations after adjustment to the local pressure and temperature conditions to solve for three-phase flow at the second location to obtain the second phase fraction, namely the liquid volume fraction. These two phase fractions along with the bulk velocity at the second location are sufficient to calculate the three-phase flow rates. The methodology is fully explained and the analytical solutions for three-phase flow measurement is explicitly provided in a step-by-step process. A Lego-like approach may be used with various sensor technologies to obtain the required measurements, although distributed acoustic sensing systems and optical flowmeters are ideal to easily and efficiently adopt the current methodology. This game-changing new methodology for measuring downhole three-phase flow can be implemented in existing wells with an optical infrastructure by adding a topside optoelectronics system.

## 1. Introduction

### 1.1. Overview

The recent developments in the distributed acoustic sensing (DAS) systems have made this promising technology viable for use in downhole multiphase flow measurement, which has been a challenging task in the oil and gas industry. The DAS system is known to measure the speed of sound (SoS) of the flowing fluid mixture because the resolution in sensor spacing is sufficient to measure fast-propagating sound waves. SoS measurement, however, is only one of the measurements required to measure two-phase and three-phase flows. The flow velocity (*V*) measurement based on either the Doppler approach or eddy-based tracking is also possible depending on the type of application and how the DAS system is installed/configured. In the present era of big data analytics and machine learning, large amounts of data produced by the powerful DAS technology need to be reduced to a manageable size for real-time monitoring of multiphase flow before implementing smart flow algorithms and methodologies that can use measurements in a combinative and coherent manner. The current work introduces a new methodology that can be used with DAS and other sound measurement technologies to measure three-phase flow.

The three-phase flow measurement methodology is based on measuring the SoS of the fluid mixture at two main locations above and below the bubble-point pressure and fluid bulk *V* at one location. The measurements that are needed to determine the three-phase flow rates are SoS at both locations, pressure/temperature (*P*/*T*) at both locations, and *V* at the second location. A Lego-like approach can be used with different sensor technologies to obtain these measurements, which are then used in a consecutive manner in two-phase and three-phase flow equations.

A full explanation of the methodology and analytical solutions for three-phase flow measurements are provided in a step-by-step approach. The same or different sensor technologies may be used simultaneously at multiple locations along the well to form flexible and custom-fit solutions. The methodology can be implemented easily and efficiently for DAS systems and optical flowmeters (OFMs), although other sensor technologies may also be used, provided those sensors can measure SoS. A developing case history involving downhole OFMs installed in a North Sea field-wide application is also discussed. This case history represents a special case of the methodology for which SoS is measured at the same location but at different times.

The new methodology has significant advantages over the traditional measurement systems and may be implemented in existing wells with an optical infrastructure by adding an appropriate topside optoelectronics hardware, which can be done even long after the start of the production.

### 1.2. Background—Downhole Multiphase Flow Measurement

There are many advantages of measuring the flow downhole, but perhaps the key point here is that it is a necessary component of production monitoring, control, and optimization of a well. For example, by measuring the flow downhole at the source and at multiple locations, it is possible to identify the zonal contribution in a multi-zone well. This constitutes the monitoring aspect of a well during the production phase, as all the information associated with the well, flow, and fluid are monitored continuously. Any change in flow, for example a water or gas breakthrough from a specific zone, is detected through monitoring. By combining this information with other intelligent completion elements such as inflow control valves (ICVs), it is possible to control (i.e., stop, regulate, etc.) the flow of fluids from that zone. Another common example for the monitoring and control aspects arises when volumetric flow rates measured by the flowmeter are low, which could be an indication to use artificial lift systems. Finally, it is also possible to optimize the production and maximize the well performance by considering all available information from all zones (e.g., [1]).

A recent categorization of flow measurement in the oil and gas industry based on the location of the measurement device places the downhole flow measurement in the most-restrictive category due to the challenging requirements associated with a harsh downhole environment, size of the device, non-intrusiveness feature, and system longevity [2]. Because of these requirements, most flow measurement technologies are not applicable for downhole deployment. In addition, most of the time, the produced fluid from the reservoir is a mixture of different phases (e.g., oil, water, gas, and sometimes even solids). Thus, the mixture could be a combination of these phases, which leads to another categorization of flow: one-phase, two-phase (e.g., oil/water, oil/gas), and three-phase (oil/water/gas) flow. The two-phase and three-phase flows are usually referred to as multiphase flows. An increase in the number of phases of a fluid mixture typically means that more measurements are needed to determine flow characteristics (e.g., *V*, *P*, and *T*) and fluid properties (e.g., density, viscosity, and SoS). It also means that multiple “deployable” technologies need to be combined using traditional and/or innovative methodologies to create coherent flow models so that multiphase flow rates can be successfully measured downhole and converted to standard conditions.

Up until two decades ago, the most common local downhole flow measurement technology in a steady-state production phase was the “old” Venturi flowmeter, the concept of which goes back 225 years. However, Venturi-type devices are typically suitable for one-phase flows and have limited flow rate ranges due to their low turndown ratios (i.e., ratio of maximum flow rate to minimum flow rate). Because these devices can measure one-phase flow only, they do not provide important measurements such as water-cut, which requires measurements of two-phase flow rates. Furthermore, the Venturi meter is not a fullbore device due to its inclined internal surface from the inlet to throat and is thus prone to erosion by the impact of solid particles (e.g., sand particles) entrained in the fluid mixture. This may result in a change in the beta ratio (i.e., ratio of throat-to-inlet diameters), causing an adverse impact on the measurement accuracy [3]. Venturi meters have been traditionally sensorized with electronic-based sensors, and the reliability/longevity of electronic sensors in harsh downhole environments has always been a concern. In particular, the measurement drift over time leads to inaccurate measurement readings. The reliability/longevity/drift concerns may be addressed by employing fiber-optic-based *P*/*T* sensors. In addition, the accuracy may be improved using a recently developed optical differential pressure (Δ*P*) sensor that measures the true Δ*P* across the Venturi inlet and throat sections with low uncertainty [4]. The Δ*P*-sensor is superior to using two independent static pressure sensors, because the static pressure sensors are designed to operate at high downhole pressures, and so the uncertainty of their measurements is usually too high for a small Δ*P* created by the fluid flow through a Venturi flowmeter. Recently, old Venturi technology was rejuvenated by integrating it with the new fiber-optic technology, which resulted in the development of an optical sibling that made use of an inverse Venturi approach [5]. The inverse Venturi, characterized by an expansion followed by a contraction of the pipe geometry, provides a fullbore continuation of the production tubing with less pressure loss. The optical Venturi flowmeter was a step to remedy the issue of measurement drift and longevity of the system to match the life of the well, but the fundamental issue—that a Venturi flowmeter alone cannot be a complete measurement system for multiphase flows—stays.

**Figure 1:**
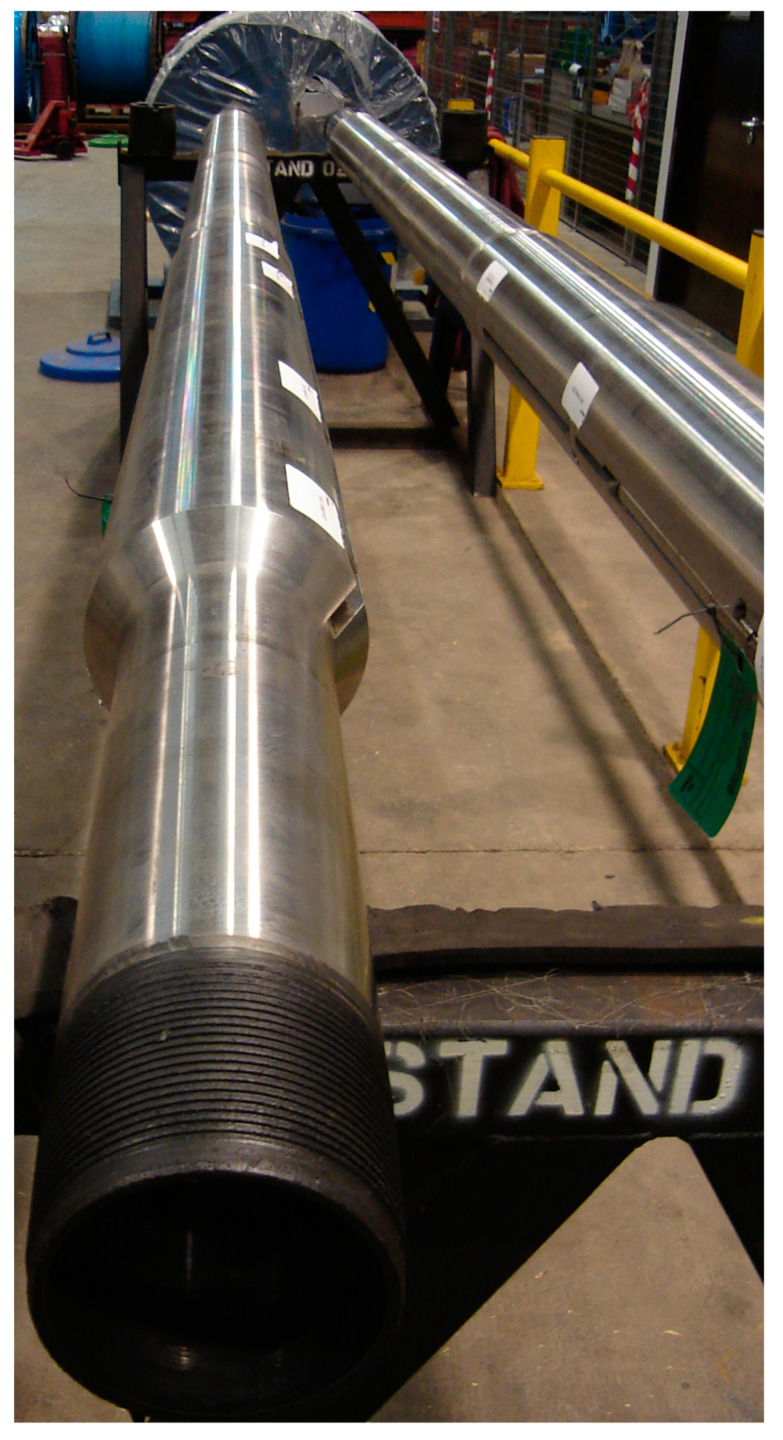
Built as a single assembly, an optical flowmeter (OFM) is a noninvasive/nonintrusive device with fullbore access and no permanent pressure loss.

The in-well OFM, shown in Figure 1, was designed for downhole real-time measurement of single-phase and multi-phase flows. It uses passive sensors externally mounted onto the pipe to sense the pressure fluctuations caused by turbulent structures and their acoustic waves propagating in the fluid medium. OFM has no optical windows or electronics downhole, and it is intrinsically safe. It is a noninvasive system because the sensors are not in contact with well fluids. It is also nonintrusive because there are no intrusions or obstructions to disturb the *V* profile, which means fullbore access and no permanent pressure loss. Two decades after its first installation in 2000 in a deepwater Gulf of Mexico [6], OFM is currently the only well-established downhole multiphase flowmeter in the traditional sense as a “local” flowmeter. The use of “local” here contrasts with the “global” or “distributed” sensing systems. OFM comes with different configurations for one-phase, two-phase, and three-phase flows. The one-phase OFM is equipped with one sensor array to directly measure the flow *V* and is typically the choice for injector applications. The multiphase OFM is equipped with two sensor arrays to measure the flow *V* and SoS of the fluid mixture [7]. SoS is an excellent indicator of volumetric fractions of oil, water, and gas phases in a fluid mixture. The sensors from both arrays are multiplexed along a single, continuous optical fiber and are interrogated from surface-installed instrumentation. The measurement is based on strain sensing using interferometric modulation [8,9]. The sensors are not coupled to the outside sleeve, thus ensuring that the OFM is unaffected by annular flow. Unlike the Venturi systems, which are optimized for unidirectional measurement, OFM measurements are bidirectional and any service change from a producer well to an injector well is a simple process [10,11]. OFM typically has a high accuracy but has some restrictions in three-phase flow measurement. For example, the use of a secondary *P*/*T* sensor is required to measure density, and this sensor is usually vertically separated from the OFM with some distance (e.g., 50 m). Furthermore, the three-phase solution is only applicable to flow conditions where the gas volume fraction (*GVF*) is in the 0–30% range. This means that there is a gap especially for horizontal wells and for flow applications where the *GVF* > 30%. To remedy this condition and to fully extend the *GVF* range, OFM can also be used in conjunction with a water-cut meter located on the surface or subsea [12,13]. This approach works well when it is a single-zone well, but when implementing for multi-zone wells, it needs to be used along with ICVs to block the zones in an alternating manner and obtain the water-in-liquid ratio (*WLR*) from the open zone. Obtaining the zonal *WLR* values discretely does not necessarily translate to the same values when all of the zones are open.

The most recent downhole monitoring technology that is also capable of measuring flow is DAS. Many companies have been founded around the DAS technology and continue to develop their own hardware and software solutions to tackle challenging problems in a wide range of industries, as well as in the oil and gas industry. A recent broad review of DAS applications from humanitarian sciences to aerospace structures and associated techniques [14] and a more specific review on the geophysics applications [15] clearly show that DAS will continue to be a prominent technology with more room to grow in the coming decades. Although DAS has been quite promising in many aspects of the oil and gas industry, including well integrity, seismic, fracture monitoring, fluid inflow detection, and flow profiling, multiphase flow measurement continues to be a challenging topic for a DAS system. A DAS system is usually capable of measuring SoS and depending on its installation/configuration and the type of application, it may also be capable of measuring flow *V* [16]. In its current state, DAS technology is still not fully explored, especially for multiphase flow measurement, for the following reasons:

- There is a lack of flow algorithms and methodologies that can utilize the measurements in a combinative and coherent approach, despite some recent efforts (e.g., [17,18]).
- The types of measurements have not been sufficient to resolve three-phase flows:
  - SoS measurement is necessary, but not sufficient, to determine the phase fractions in a three-phase flow.
  - *V* measurement in a DAS system is not always possible, particularly when the fiber cable is clamped onto the tubing as a straight line (DAS_line_). A Doppler-based *V* based on SoS measurement is confidently obtained from gas flows, but in liquid flows, the uncertainties could be very high [19]. The flow *V* may be measured with specific configurations such as circularly-wrapped and closely-distanced fiber sensors (DAS_coil_), a configuration that is not typical to DAS [20,21]. Derivatives of this specific configuration may use other special arrangements including the use of bare fibers (i.e., without fiber coating), which resemble the in-well OFM.
- The amount of data acquired in a DAS system for the complete length of fiber usually adds up to large sizes and thus a selective process and reduction of data are required.
- The data are usually post-processed and not reported in real-time.

There are also other fiber-optic sensing technologies that can make complementary measurements that can be used in the prediction of multiphase flow rates. These distributed fiber-optic sensing technologies include distributed temperature sensing (DTS), distributed pressure sensing (DPS), and distributed temperature and strain sensing (DTSS). Recent reviews of the use of these technologies in oil and gas applications and how these sensor technologies are categorized based on their measurement approaches are available in the public domain (e.g., [22]).

### 1.3. Objectives

The current work introduces a new methodology [23,24] for downhole three-phase flow measurement using various sensors and measurement technologies that are capable of measuring fluid bulk *V* at one location and SoS of the fluid mixture at multiple locations above and below the bubble-point pressure (*P*_b_). For some special cases where the pressure at the single measurement location is fluctuating around *P*_b_, one main measurement device may be sufficient to resolve the three-phase flow by using its own measurements at different times: the two-phase flow solution corresponding to the time when the pressure is above the *P*_b_ provides the feedback to the three-phase solution when the pressure is below the *P*_b_, thereby creating a feedback cycle between the two flow solution models.

Fiber-optic sensors and associated technologies can efficiently adopt this methodology, although it is also possible to utilize electronic sensors or a hybrid system as the methodology is independent of the sensor type. This methodology can be implemented to create custom-fit solutions for different production scenarios for new wells, but also can be retrofitted to existing wells with an optical infrastructure.

## 2. New Methodology

**Figure 2:**
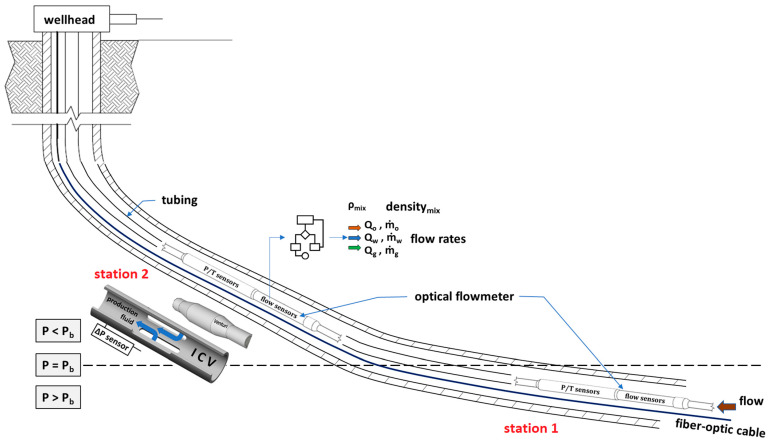
New three-phase flow measurement methodology requires measuring speed of sound (SoS) around bubble-point pressure (*P*_b_) along the well at Stations 1 and 2 using same sensor technology multiple times or a combination of various sensor technologies at multiple locations. ICV: inflow control valve; Q: volumetric flow rate; ṁ: mass flow rate; o, w, g: oil, water, gas, respectively.

The three-phase flow measurement methodology is based on the measurements of SoS at different locations along the well where the pressure is above the bubble-point pressure (*P* > *P*_b_) at the first location (Station 1) and *P* < *P*_b_ at the second location (Station 2). A bulk *V* measurement is also necessary at one of the locations, preferably at the second location. These measurements combined with *P*/*T* measurements will allow the calculation of the phase flow rates in a three-phase flow (Figure 2). The fluid flow at the first location where *P* > *P*_b_ represents presumably a two-phase flow (if not one-phase flow) because the gas is dissolved in the oil phase. SoS measurement at this first location will yield the phase fraction for the two-phase flow (e.g., water cut) and this information is used when solving the three-phase flow equations at the second location where *P* < *P*_b_. The SoS measurement at the second location along with the phase fraction from the first location will yield the other phase fraction (e.g., liquid volume fraction) at the second location. Using these two phase fractions and the measured bulk *V* at the second location, the phase flow rates may be determined. The details of the methodology will be discussed later using a step-by-step process.

### 2.1. Sensor Configurations

A sample list of major measurements from various sensor technologies (Table 1) may be used with the new methodology. By using various sensor technologies at two stations along the well, it is possible to predict the three-phase flow rates. This can be achieved in different ways: by using the same sensor technology at both stations or combining various sensor technologies at each station. Some of the major sensor configurations for the three-phase flow measurement are listed in Table 2 below. The minimum required measurements for using the new methodology are SoS at both stations (SoS_1_ and SoS_2_), *P*/*T* values at both stations (*P*_1_, *P*_2_, *T*_1_, and *T*_2_), and the bulk *V* at Station 2 (*V*_2_). A Lego-like approach may be used with these sensor technologies to obtain the minimum required measurements. All of these measurements will then need to be combined in a coherent flow solution model so that the phase flow rates can be calculated.

A detailed description of how these measurements are used to determine the three-phase flow rates is provided later. Here, the key configurations in Table 2 and their possible extended versions are summarized:

- *Configuration 1* is an all-DAS solution at both stations. Using the DAS_coil_ configuration, *V* measurements are also possible. Because only one *V* is sufficient, preferably at Station 2, the DAS configuration at Station 1 could be DAS_line_ (i.e., no need for *V*_1_ measurement). While a DAS system measures SoS and *V*, *P* and *T* may be measured by a *P*/*T* sensor. In an ideal configuration, only one optical fiber may be sufficient for a DAS system and *P*/*T* sensors at both stations.
- *Configuration 2* is an all-distributed-sensing solution using DAS_line_ and DAS_coil_ for SoS and *V* measurements, DTS for temperature measurements at both locations, and DPS for pressure measurements at both locations.
- *Configuration 3* is one of the ideal solutions that combines the power of OFM and DAS technologies. The solution is possible using one single optical fiber: a DAS_line_ system and a *P*/*T* sensor at Station 1 measure SoS_1_, *P*_1_, and *T*_1_, while the OFM measures SoS_2_, *P*_2_, *T*_2_, and *V*_2_ at Station 2.
- *Configuration 4* uses DAS_line_ and *P*/*T* sensors at both stations, while *V*_2_ at Station 2 is measured by a single-phase Venturi flowmeter.
- *Configuration 5* uses DAS_line_ and *P*/*T* sensors at both stations, while *V*_2_ at Station 2 is measured by a Δ*P*-gauge that measures Δ*P* between two different cross-sectional areas at Station 2. A Δ*P*-gauge may use a single “true” Δ*P* sensor or may operate based on two separate *P* sensors placed across different cross-sectional areas.
- *Configuration 6* is an all-OFM solution at both stations. At each station SoS, *V*, *P,* and *T* are measured. A more practical case is when the OFMs are installed in a multi-zone well. By blocking the upper zone production first, the phase flow rates at the upper station produced by the lower zone may be obtained. The upper zone production is then restarted, and an estimate of the upper zone phase flow rates can be made based on all of the available information.

In the event that the exact location of *P*_b_ changes relative to its initial location in the well, let us say due to the changes in the reservoir characteristics, then the sensor locations may be adjusted easily in a DAS system because DAS is a flexible system where any part of the fiber can be allocated as a sensor. This would be more problematic for a local measurement system such as OFM, as it is part of the production tubing at its originally installed location. Therefore, if the system consists of two OFMs installed at Stations 1 and 2 (i.e., *Configuration 6*), the recommendation is then to install the OFMs at locations comfortably satisfying the pressure conditions (i.e., for Station 1, *P* >> *P*_b_, and for Station 2, *P* < *P*_b_). If the system consists of a DAS system and an OFM (i.e., *Configuration 3*), DAS should be allocated as the Station 1 sensor and OFM should be installed as the Station 2 sensor, as already stated in Table 2.

### 2.2. Solution Domain

**Figure 3:**
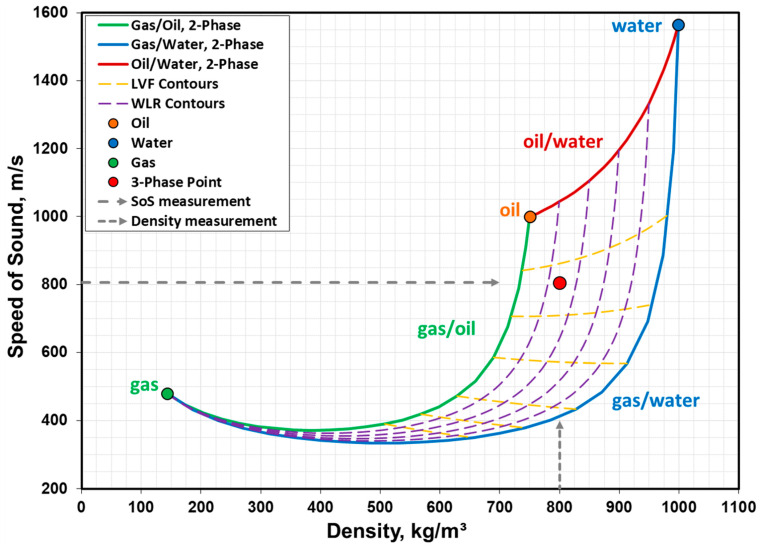
The solution envelope for a three-phase flow can be mapped by contours of liquid volume fraction (*LVF*) and water-in-liquid ratio (*WLR*), and is bounded by two-phase flow curves of oil/water, oil/gas, and gas/water. The *WLR* contours from the oil phase to water phase represent 20%, 40%, 60%, and 80% water fractions, whereas the *LVF* contours from the liquid phase to gas phase represent 98%, 95%, 90%, 80%, 70%, and 60% liquid fractions. The three-phase solution point can be reached by external measurements of speed of sound (SoS) and density, and this corresponds to a specific pair of *LVF* and *WLR*. The boundary conditions for crude oil, methane-rich natural gas, and production water are from a North Sea field at *P* = 175 bar and *T* = 90 °C.

First, let us consider an SoS measurement system and discuss the solution envelope in the (density, SoS) domain (Figure 3). The boundaries of the solution envelope represent the two-phase solutions of oil/water, oil/gas, and gas/water.

The boundaries in Figure 3 can be obtained using the mixture density based on volumetric phase fractions (Equation (1)) and Wood equation (Equation (2), [25]):

(1) $$\rho_{m}=\left(1-\phi \right)\rho_{1}+\phi \rho_{2}$$

(2) SoSm=1−ϕρ1+ϕρ21−ϕρ1a12+ϕρ2a22−12

where

*ρ_m_*: density of mixture;
*ρ*_1_: density of phase 1;
*ρ*_2_: density of phase 2;
*SoS_m_*: SoS of mixture in the infinite medium;
*a*_1_: SoS of phase 1 in the infinite medium;
*a*_2_: SoS of phase 2 in the infinite medium;
*ϕ*: phase fraction between two phases [0–1].

Wood [25] stated that Equation (2) is applicable to a homogeneous mixture of any two fluid media that do not react chemically and have no resonant motion. By systematically changing the phase fraction *ϕ* from 0 to 1 with a reasonable resolution, we obtained the (*ρ_m_*, SoS_m_) pairs. When *ϕ* = 0 (i.e., 100% phase 1), this corresponds to the pair (*ρ*_1_, *a*_1_); when *ϕ* = 1 (i.e., 100% phase 2), this corresponds to the pair (*ρ*_2_, *a*_2_). Using this process, between phases 1 and 2, 2 and 3, and 1 and 3, we obtained the two-phase flow boundaries of the solution envelope in Figure 3. The phases of 1, 2, and 3 arbitrarily represent oil, water, and gas, respectively. Thus, the pairs (*ρ*_o_, *a*_o_), (*ρ*_w_, *a*_w_), and (*ρ*_g_, *a*_g_) represent the one-phase oil, one-phase water, and one-phase gas conditions, respectively (i.e., the corners of the three-phase solution envelope). Note that in the case of gas/liquid boundaries, the curves have minimum SoS regions due to the impact of suspended gas bubbles on acoustic propagation in liquid.

The three-phase flow solution envelope bounded by the two-phase flow curves may further be mapped by the contours of *WLR* and the liquid volume fraction (*LVF*). The *WLR* contours intersect the oil/water two-phase flow solution, whereas the *LVF* contours run parallel to the two-phase flow oil/water boundary and across the two gas/liquid flow boundaries (i.e., gas/oil and gas/water). While the boundaries of the three-phase flow envelope are obtained using the two-phase flow versions of the density and SoS expressions, as demonstrated by Equations (1) and (2), the contours of *LVF* and *WLR* require the more general versions of Equations (1) and (2) involving all three phases. We then need to modify Equations (1) and (2) for a three-phase flow representation:

(3) $$\rho_{m}=\left(1-WLR\right)\left(LVF\right)\rho_{o}+(WLR)\left(LVF\right)\rho_{w}+\left(1-LVF\right)\rho_{g}$$

(4) SoSm=ρmLVF1−WLRρoao2+LVFWLRρwaw2+1−LVFρgag2−12

The detailed derivations of Equations (3) and (4) are provided in Appendix A. The *LVF* and *WLR* contours can now be plotted by keeping one of them at a constant value (i.e., at the “contour” value) between 0 and 1 and varying the other with a reasonable resolution from 0 to 1. This allows us to determine the (*ρ_m_*, SoS_m_) pairs at constant contour values.

We can now focus on the three-phase solution point inside the three-phase solution envelope represented by the red marker. This three-phase point corresponds to a specific (*LVF*, *WLR*) pair. It also corresponds to a specific (*ρ_m_*, SoS_m_) pair. Thus, the three-phase point is associated with four parameters that are related through Equations (3) and (4). Knowing two of the four parameters will suffice for solving the system completely because then Equations (3) and (4) form a two-equation/two-unknown system. Note that SoS_m_ refers to the mixture SoS in the infinite medium, whereas the external SoS measurement is based on the SoS measurement in the pipe. The relation between the two is given by the Korteweg-Lamb equation, Equation (5) [26].

(5) SoSm=1apipe2−ρmdt1−ν2E−12

where

*a_pipe_*: SoS of mixture in the pipe (measured by sound measurement system);
SoS_m_: SoS of mixture in the infinite medium (used interchangeably with *a_m_*);
*d*: pipe diameter;
*t*: pipe wall thickness;
*E*: modulus of elasticity of pipe material;
*ν*: Poisson ratio of pipe material (0.3 for rigid bodies such as steel).

It is possible to solve for the measured SoS, *a_pipe_*, by using Equation (5) in Equation (4) and rearranging as in Equation (6).

(6) apipe=ρmLVF1−WLRρoao2+LVFWLRρwaw2+1−LVFρgag2+dt1−ν2E−12

If the (*ρ_m_*, SoS_m_) measurement is made by an external system, the phase fractions *LVF* and *WLR* will be solved using the two-equation/two-unknown system (i.e., Equations (3) and (6)).

Note that *a_pipe_* measured by the sound measurement system does not depend on the *V* of the fluid mixture, because it is obtained by taking the average of the SoS measured in both directions thereby removing the effect of *V* on the measured SoS.

In the application of Equation (6) to a pipe, we are interested in the planar acoustic waves propagating within the pipe along the pipe axis. The reason is that the typical sensors around the pipe (in OFM or DAS systems) detect the radial impact of sound waves on the pipe wall as the sound waves propagate through multiple sensor locations along the pipe axis. Planar wave assumption requires that the wavelengths are typically larger than the pipe diameter. As a result, the acoustic wavelengths and frequencies of interest must be consistent in the analysis of the signals obtained from the sensors. The distribution of the phases within the fluid mixture is also important and this is considered under flow patterns associated with the macro-motion of the fluid flow. The key question is whether or not the SoS measurement represents the SoS of the fluid mixture, as the solution domain shown in Figure 3 makes this assumption. The discussion of how the flow patterns impact the SoS measurement is provided in detail later in Section 2.4.

### 2.3. Solution Method

**Figure 4:**
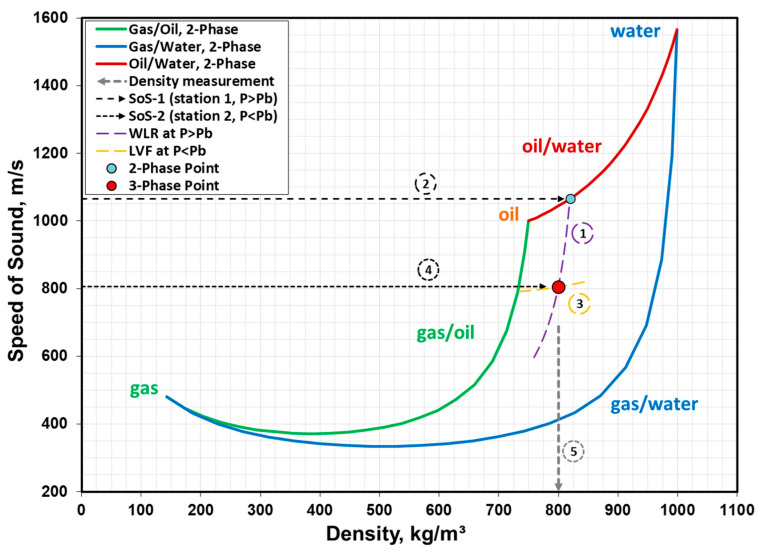
The three-phase measurement methodology requires a two-step approach: first, measuring the speed of sound (SoS) of a mixture at *P* > *P*_b_ and determining the water-in-liquid ratio (*WLR*), then measuring the SoS of the mixture at *P* < *P*_b_ and combining it with the *WLR* to determine the liquid volume fraction (*LVF*). Contour 1: *WLR* at Station 1; Line 2: SoS_m_ at Station 1; Contour 3: *LVF* at Station 2; Line 4: SoS_m_ at Station 2; Line 5: density at Station 2.

A step-by-step description of the current methodology is provided below:

- *Step 1:* A first SoS measurement is made at a depth along the well (Station 1) where the pressure is above the bubble-point pressure, *P* > *P*_b_, and thus with no free gas in the fluid mixture. This is the SoS measurement at *P*_1_ and *T*_1_ made in the pipe (i.e., *a_pipe_* in Equation (5) or Equation (6)) by the sound measurement system and carries the compliance effects of a closed conduit.
- *Step 2:* The next step involves using *LVF* = 1 (i.e., no free gas) and rewriting *ρ_m_* in Equation (3) and SoS_m_ in Equation (4) as a function of *WLR* only. All the individual phase properties (*ρ_i_* and *a_i_* values) in Equations (3) and (4) are obtained from tabulated values for *P*_1_ and *T*_1_ measured at Station 1.
- *Step 3:* The expressions obtained for *ρ_m_* and SoS_m_ are used in Equation (5) along with the SoS measurement in the pipe, *a_pipe_*, resulting in a quadratic equation with a single unknown (*WLR*). The solution of the quadratic equation for *WLR* is explained in detail in Appendix A. This *WLR* represents the two-phase flow point at the intersection of the oil/water two-phase flow boundary and the *WLR* contour (Contour 1) in Figure 4. Once *WLR* is solved, the corresponding *ρ_m_* is calculated using Equation (3) and the corresponding SoS_m_ is calculated using Equation (4) or Equation (5). Line 2 represents the SoS_m_ for the infinite medium at *P*_1_ and *T*_1_ (i.e., Station 1).
- *Step 4:* A second SoS measurement is made at a depth (Station 2) where the pressure is below the bubble-point pressure, *P* < *P*_b_, and thus with free gas present in the fluid mixture. This is the SoS measurement at *P*_2_ and *T*_2_ made in the pipe (i.e., *a_pipe_* in Equation (5) or Equation (6)) by the sound measurement system and carries the compliance effects of a closed conduit.
- *Step 5:* The next step involves using the newly-found *WLR* in Step 3, adjusting it for *P*_2_ and *T*_2_ conditions, and rewriting *ρ_m_* in Equation (3) and SoS_m_ in Equation (4) as a function of *LVF* only. The individual phase properties (*ρ_i_* and *a_i_* values) in Equations (3) and (4) are obtained from tabulated values for *P*_2_ and *T*_2_ measured at Station 2.
- *Step 6:* The expressions obtained for *ρ_m_* and SoS_m_ are used in Equation (5) along with the SoS measurement in the pipe at Station 2, *a_pipe_*, resulting in a quadratic equation with a single unknown (*LVF*). The solution of the quadratic equation for *LVF* is explained in detail in Appendix A. The *LVF* contour is represented by Contour 3 and the intersection of the (*LVF*, *WLR*) pair represents the three-phase flow point in Figure 4. The (*LVF*, *WLR*) pair is used to calculate the corresponding SoS_m_ (Line 4) via Equation (4) and the corresponding *ρ_m_* (Line 5) via Equation (3). Line 4 represents the SoS_m_ for the infinite medium at *P*_2_ and *T*_2_ (i.e., Station 2).

**Figure 5:**
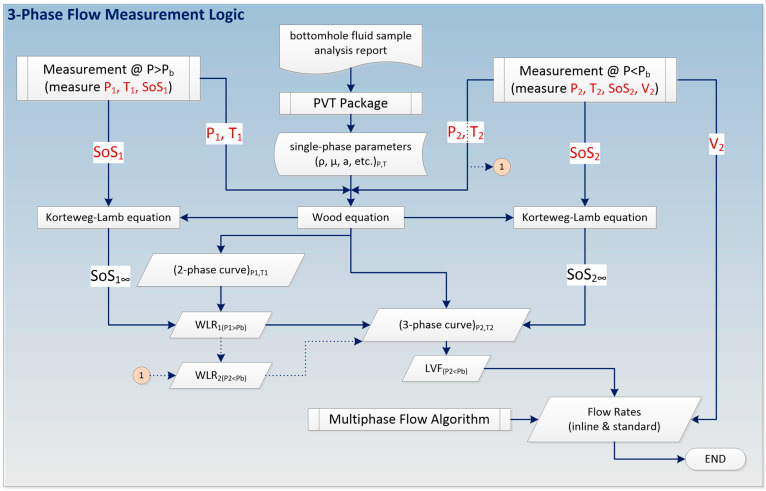
The three-phase measurement logic involves the use of speed of sound (SoS) measurements and the Wood equation from which phase fractions are determined, and by using a multiphase flow algorithm, the flow rates are calculated. Connection point 1 refers to the process of converting *WLR*_1_ to *WLR*_2_ at *P*_2_ and *T*_2_ conditions.

The three-phase flow measurement diagram is provided in Figure 5 and is described in detail below. A bottomhole fluid sample analysis along with a pressure-volume-temperature (*PVT*) package may be used to create single-phase fluid properties as a function of *P* and *T*. These fluid properties include density, viscosity, SoS, and formation volume factors of individual phases and are prepared in tabulated form for a range of *P* and *T* values that cover the range of the specific application. For each *P* and *T* measurement, the fluid properties are interpolated using the table values, which are kept in the topside flow computer.

*P*_1_, *T*_1_, and SoS_1_ are measured at the first measurement station where *P*_1_ > *P*_b_. *P*_1_ and *T*_1_ are also used to determine the single-phase properties and the solution envelope of the application is created. The single-phase properties along with SoS_1_ may then be used in the Wood and Korteweg-Lamb equations to determine the *WLR* at Station 1. In parallel to the first measurement station, *P*_2_, *T*_2_, SoS_2_, and *V*_2_ are measured at the second measurement station where *P*_2_ < *P*_b_. *P*_2_ and *T*_2_ are used to determine the single-phase properties and a slightly different solution envelope of the application is created. This time, the single-phase properties along with the SoS_2_ and the *WLR* obtained earlier (after adjustment to local *P*_2_ and *T*_2_ conditions) may be used in Wood and Korteweg-Lamb equations to determine the *LVF* and the three-phase flow solution point at Station 2.

There may be a slight difference between the *WLR* values obtained at Stations 1 and 2 due to the difference in pressure. If the stations are too far apart and *P*_2_ is significantly lower than *P*_b_ at Station 2, it is possible to consider the variation in *WLR* using a first-order linear interpolation along the pressure interval between the two stations, as explained in detail in earlier works [2,27]. This process of converting *WLR*_1_ (*P*_1_ > *P*_b_) to *WLR*_2_ (*P*_2_ < *P*_b_) is shown by the dotted line and the connection point 1 in Figure 5. Note that as the fluid mixture moves from Station 1 to Station 2, a small amount of gas is liberated from the oil while the water volume is practically the same, and thus a slight increase in *WLR* is expected. However, the key point is that the impact of introducing gas into an oil/water two-phase mixture is dramatically greater than the impact of the relative increase in water in the oil/water/gas three-phase mixture. This can be observed from Figure 3: when gas is introduced, the change in SoS value is dramatic along the nearly-vertical *WLR* contours. On the other hand, when *WLR* is slightly increased, the change in SoS value is not so significant along the nearly-horizontal *LVF* contours.

Once the (*LVF*, *WLR*) pair as well as the mixture density are determined, the inline volumetric and mass phase flow rates may be calculated using the measured *V*_2_ and the cross-sectional area at the second station. It is also possible to implement various multiphase flow algorithms to consider possible slip conditions between the phases. These are discussed in detail in Appendix A.

It should be noted that the main goal of the current methodology is to measure three-phase flow at one location using a device that is normally not capable of measuring three-phase flow. This is achieved by feeding the measurement of an additional device measuring the same flow at an early stage when it has not yet developed to a three-phase flow. The measurement by the additional device when the flow is two-phase (i.e., at Station 1) is the key because it provides the entry point (*WLR* contour) to the three-phase flow solution envelope shown in Figure 4. Consequently, this methodology monitors the development of the three-phase flow from its early stages. As briefly mentioned earlier in the introduction, other options exist to measure three-phase flow downhole directly using sensors located in the three-phase flow section of the tubing, but these options typically have limitations. For example, SoS and *V* measurements by an OFM or DAS system can be combined with a secondary *P*/*T* gauge along the length of the well located some distance away from the main sensor system. A density prediction is possible based on the pressure loss due to the frictional force and weight of the body of the fluid mixture between the pressure measurement points. This has been demonstrated and implemented in the field in earlier works [28,29]. However, the three-phase flow solution was only applicable to flow conditions when *GVF* was in the 0–30% range. Above this range, the three-phase flow solution envelope starts converging, and the small *LVF* makes it extremely challenging to distinguish the oil and water phases without the prior knowledge of *WLR,* unlike the current methodology.

The implication of measuring *WLR* at Station 1 is of great importance, because this not only allows for the implementation of this methodology to measure three-phase flow in its typical use by combining the measurements above and below *P*_b_, but it also extends the three-phase flow solution envelope to flow conditions where the *GVF* > 30%. This can be visualized by monitoring the three-phase flow solution point, which currently resides in the high *LVF* (i.e., low *GVF*) region in Figure 3. As *GVF* increases, the gas/oil and gas/water boundaries of the three-phase flow solution envelope start converging towards the left of the envelope. This region represents the gas-rich and small *LVF* region within which it is extremely challenging to distinguish the oil and water phases. This would not be an issue with the current methodology, because the *WLR*_2_ at Station 2 is available through the adjustment of *WLR*_1_ at Station 1 for the *P*_2_ and *T*_2_ conditions.

### 2.4. Measurement Accuracy and Uncertainty

The methodology described in this work is based on an analytical approach and the solutions are obtained via second-order quadratic equations (Appendix A). The measurement accuracies for phase flow rates depend on the individual measurements of SoS, *V*, *P*, and *T* by the different measurement systems, exemplified in Table 1 and Table 2. However, the boundary conditions (BCs) of the solution domain, namely, SoS of individual phases (oil, water, and gas) at the local *P*, as well as *T* conditions, are equally important. These BCs will determine how the solution envelope is positioned in the density/SoS domain (Figure 3). If the BCs are not accurately determined, then the phase fraction information (*LVF*, *WLR*) obtained from the solution envelope will be in error, even if the devices provide precise measurements of SoS, *V*, *P*, and *T*.

The typical approach is to determine these BCs as a function of *P* and *T* from bottomhole fluid sample analysis. However, sometimes, a sample analysis may not necessarily provide the true values for a number of reasons, including potential errors in sampling activities [30] and laboratory-related issues. In such cases, there may be options to determine these BCs by using surface measurements [2,27]. Furthermore, sometimes, BCs may be a direct measurement from the device. For example, a frequently seen scenario is that a well may be producing dry oil in its early stages, after which there may be a water breakthrough. In those early stages, the SoS measured by the measurement system represents a direct measurement of single-phase oil SoS.

An inspection of the solution envelope in Figure 3 provides clues on strong and weak points associated with three-phase flow measurement of this type. The liquid-rich region represents the high-resolution part of the solution domain. With the *WLR* information known, the mixture SoS measurement by the device uses a unique three-phase flow solution point corresponding to the actual flow conditions. As *GVF* increases, the parabolic gas/liquid boundaries of the solution domain starts converging and the region around the minimum values of the parabolic curves becomes critical for several reasons. First, the SoS measurement by the device may correspond to two solutions: one for a gas-rich fluid mixture and the other for the liquid-rich mixture. Consequently, prior knowledge of the field (e.g., gas producer or oil producer) will help determine the correct solution. Second, the region around the minimum values corresponds to a wide density range, while the range of SoS values is narrow. A slight error in SoS measurement may move the solution point significantly in the solution domain. Thus, the uncertainty of SoS measurement plays a critical role in the accuracy of phase flow rates. With increased *GVF* values, slug flows may also develop in this region and the flow becomes intermittent. When the phases in the fluid flow are not mixed well, the SoS value measured by the device is not a good representative SoS of the fluid mixture. As such, it should not be used in conjunction with the solution envelope to determine the phase fractions because the boundaries of the solution envelope are based on the Wood equation under well-mixed fluid flow conditions. The determination of slug flow and its severity may typically be obtained from the standard deviation of the SoS measurement. For well-mixed fluid flows, the standard deviation of a SoS measurement is expected to be very stable (e.g., 0.5% or less), whereas for slug flows, the standard deviation may be at least an order of magnitude higher or more. Slug flow, by its characterization, consists of slug units composed of bullet-shape gas pockets (Taylor bubbles) and plugs of liquid slugs [31,32]. The gas pockets occupy a large portion of the cross-sectional area of the pipe, while the liquid slugs carry distributed gas bubbles and separate the gas pockets. As a result, the SoS measured by the sensors may jump between the SoS of the individual phases (e.g., ~400 m/s for gas SoS and above 1000 m/s for oil/water mixture SoS), dramatically increasing the standard deviation of the SoS measurement by the device.

The current methodology may be applied to a group of different sensor technologies. These sensor groups typically have different measurement uncertainties and users should make their own assessment of how these uncertainties will impact the overall uncertainty of the system. For example, OFM technology has shown that once BCs are optimized, *WLR* measurements are within ±1% of the separator measurements and phase flow rates are well within ±5% [2,27].

The deviation of the well also plays a critical role in the implementation of the current methodology, which assumes that there are three key depths: the deepest measurement location, defined as Station 1, where *P* > *P*_b_ with no gas present; the critical depth where *P* = *P*_b_; and the location above the critical depth where *P* < *P*_b_ with gas present. For vertical and deviated wells, these key depths can clearly be identified, while for horizontal wells, it is necessary to carefully determine those specific locations where the pressure conditions are satisfied for each station. When determining those locations, an important requirement should be the measurability of SoS of the fluid mixture. The SoS measurement must be a good representative of a well-mixed flow as the three-phase flow solution envelope is based on well-mixed flows. Based on laboratory and field experiences, SoS measurement is typically a good representative of the three-phase fluid mixture when the flow patterns are bubbly, annular, or disperse. As mentioned above, slug flows are problematic because the SoS measured by the sensors may jump between the SoS of the individual phases. Taking these points into account, the use of the current methodology can be optimized for use with different well deviations, from horizontal to vertical. For the locations where *P* < *P*_b_, gas will start coming out of the oil phase. The amount of gas will be determined by the deviation of pressure from *P*_b_. In its ideal implementation for vertical and moderately-deviated wells, Station 2 measurement will not be too far from the critical depth of *P* = *P*_b_. A DAS system, for example, can be used to interrogate a portion of the fiber length not too far from the critical depth. With only a limited amount of gas, the flow pattern of the fluid mixture is expected to be bubbly flow, which is a suitable flow pattern for a reliable SoS measurement of the fluid mixture. Likewise, for horizontal and near-horizontal wells, it may be necessary to conduct a flow pattern estimation study for the application to determine the best location for Station 2, so that a reliable SoS measurement is possible.

### 2.5. Special Case

In a special case, this methodology may be used with a single device at a single location if the pressure, *P*, at the device location fluctuates around *P*_b_, such that *P*_t2_ ≤ *P* ≤ *P*_t1_, where t1 refers to a specific time when *P*_t1_ > *P*_b_, and t2 refers to a specific time when *P*_t2_ < *P*_b_. In such circumstances, a single device, measuring SoS and *V* at a single location, is sufficient because the device measurements at different times may be used to resolve three-phase flow rates. When *P* = *P*_t1_ > *P*_b_, the flow is a two-phase flow, and the device location effectively functions as Station 1 at time t1. The *WLR* is calculated from the two-phase flow solution curve, and the device continues to report two-phase in-situ flow rates. When *P* = *P*_t2_ < *P*_b_, the flow is a three-phase flow, and the device location effectively functions as Station 2 at time t2. This triggers the current methodology such that the previous *WLR* measurement from Station 1 (i.e., t1) is used as an input to the measurements at Station 2 (i.e., t2). With the known *WLR* from Station 1, the other phase fraction *LVF* can be determined using the three-phase flow solution domain as described earlier. The device then reports in-situ and standard phase flow rates for three-phase flow.

### 2.6. New Solution Features

The methodology introduced in the current work provides an efficient approach to measure three-phase flow downhole for a measurement system or a combination of systems that can be strategically positioned in the well. Accordingly, a distributed system (e.g., DAS) that can provide measurements on any section of the fiber-optic cable along the well would be an ideal choice for the implementation of this methodology. There are also significant advantages of using this multiphase measurement methodology over the traditional methods. Some of these advantages are listed below:

- *3-Phase flow measurement:* This methodology allows for three-phase flow measurement by utilizing multiple SoS measurements along the well by using the same sensor technology at multiple locations or combining different sensor technologies.
  – A special case may occur in the field when the pressure at the device location fluctuates around *P*_b_ (i.e., *P*_t2_ ≤ *P* ≤ *P*_t1_, where *P*_t1_ > *P*_b_ and *P*_t2_ < *P*_b_). In this case, a single device, measuring SoS and *V* at one location, may be used at different times to resolve the three-phase flow.
  – The three-phase flow solution extends to flow conditions where the *GVF* > 30%. As *GVF* increases, the gas/oil and gas/water boundaries of the three-phase flow solution envelope start converging, and the small *LVF* makes it extremely challenging to distinguish the oil and water phases. This would not be a problem with the current methodology, because the *WLR*_2_ at Station 2 is available through the adjustment of *WLR*_1_ at Station 1 for the *P*_2_ and *T*_2_ conditions.
- *Independent of sensor type:* This method is independent of the sensor type as long as the sensors measure SoS, although the ideal systems are DAS systems and OFMs.
- *Retrofit solutions for wells with fiber-optic infrastructure:* The current methodology can be applied to existing wells with a fiber-optic infrastructure by adding an appropriate topside optoelectronics system.
- *Nonnuclear 3-phase solution:* The three-phase flow measurement has no nuclear-based measurement such as gamma densitometers, so there are no regulatory concerns.
- *High turndown ratio:* In its typical use with DAS systems and/or OFM systems, there is no limitation in high fluid velocities, which translates to a high turndown ratio when compared with Venturi-based systems.
- *Minimum pressure loss:* In its typical use with DAS systems and/or OFM systems, the pipe geometry is fullbore and there is no pressure loss when compared with Venturi-based systems.
- *Better economics:* In most cases with a fiber-optic infrastructure, the current methodology requires minimum or no additional equipment when a DAS system is already in use and the bulk *V* measurement is available.

## 3. Developing Case History

One operator in the North Sea region has been a pioneer in using optical measurement technologies by installing a large number of downhole OFMs in three separate fields since 2006. The development of the fields, the selection of the monitoring systems, how the monitoring systems are strategically expanded as more wells are added to the fields, and the operating principles of the OFM have been discussed in detail in an earlier work [2] and will not be discussed here again.

The motivation to include this developing case history in the current work is to demonstrate that the methodology may be applied to actual field conditions by either implementing the special solution (not a rare situation after all) or retrofitting the complete solution to the existing optical infrastructure.

### 3.1. Current Status of Wells

The number of OFM installations in the three fields has reached approximately 70. Each well has one OFM installed at a location where the pressure is greater than the bubble-point pressure (*P* > *P*_b_). At this pressure, the gas is dissolved in the oil phase, and so there is no free gas in the fluid medium. The flow is two-phase, and the fluid is a mixture of oil and water. Recently, the pressure in some of the wells started to decrease intermittently below the bubble-point pressure (*P* < *P*_b_), resulting in the liberation of gas from the oil phase intermittently. With the presence of gas as the third phase, the flow becomes a three-phase flow for those short durations. As a result, the two-phase OFM is exposed to a three-phase flow intermittently. During the time intervals when *P* < *P*_b_, the measurement accuracy is expected to be affected adversely, because OFM’s existing algorithm flags are typically set to two-phase oil/water flow. Generic descriptions of the wells that are excellent candidates to experience this sort of behavior are provided in Table 3, along with the predicted bubble-point pressures, recent pressure ranges reported by the OFMs, potential low pressures predicted by the operator, as well as the equipment installed above and below the OFMs (which could be important for sound characteristics of the wells).

### 3.2. Future Work

It is expected that at some point in the future, the three wells in Table 3 will experience a behavior such that the pressure at the measurement location fluctuates around *P*_b_. This means that the fluid flow going through OFM will be alternating between two-phase and three-phase flows. The methodology described in the current study may then be implemented using two different potential approaches:

- Implement the special case described earlier. The single device (i.e., the OFM) will measure SoS and *V* at different times to resolve the three-phase flow: solution of two-phase flow equations when *P* > *P*_b_ will provide the *WLR* information that will then be used as an input for the three-phase flow equations when *P* < *P*_b_. The feedback cycle will continue as the pressure fluctuates around *P*_b_. This approach represents the most cost-effective solution as it does not require a hardware addition to the existing system. The methodology may be implemented in the flow algorithm of the flow computer located topside. However, this solution should still be seen as a temporary solution, because as the local pressure starts deviating from *P*_b_, the accuracy of the measurements will be in question (i.e., broken feedback cycle).
- A more permanent solution is to introduce a DAS system on the same OFM fiber line or using a separate fiber. DAS can measure SoS at a deeper location (i.e., Station 1) where *P* > *P*_b_, while OFM provides measurements for the upper location (i.e., Station 2) where *P* < *P*_b_.

Implementing one of these approaches will be sufficient to solve the three-phase flow rates. The selection process requires a decision by the operator. Eventually, these flow rates obtained by any of these solutions will be compared with the well test data to determine the performance of the system.

### 3.3. Variation of Water-in-Liquid Ratio (WLR)

One of the key points of this methodology is the variation of *WLR* between the measuring stations. This is important because we are using the *WLR* information obtained at Station 1 for solving the *LVF* at Station 2. So, we are naturally interested in how the *WLR* varies as the fluid mixture moves up.

**Figure 6:**
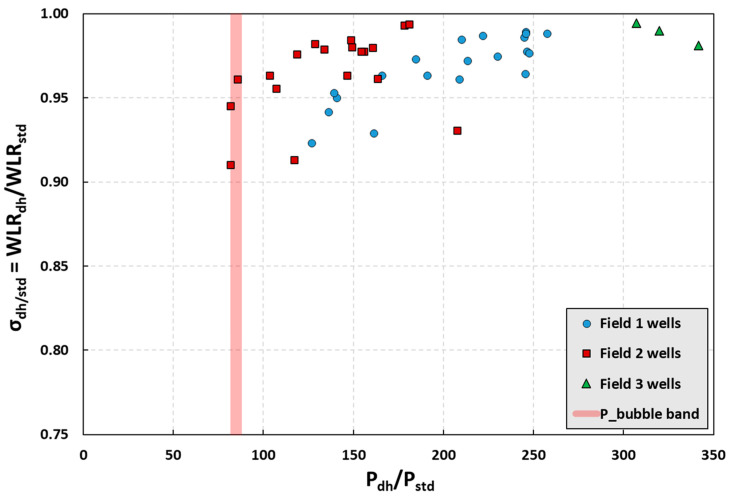
As the fluid mixture moves from reservoir to the surface, the ratio of downhole water-in-liquid ratio (*WLR*) to standard *WLR* stays greater than 0.90 for all wells in the three Northern Sea fields and greater than 0.95 for the majority of the wells.

The most important factor in the variation of *WLR* is pressure. As the pressure decreases from reservoir to surface, gas emerges from the oil phase, the oil volume decreases, and *WLR* starts to increase, reaching its largest value at the surface. The variation of *σ*_dh/std_ (defined as the ratio of downhole *WLR* to standard *WLR*) with normalized pressure is provided in Figure 6 for 30 wells from three different fields with multiple data points from some of the wells. For each of these wells, *WLR*_dh_ is at the downhole pressure measured by the OFM and *WLR*_std_ is at the standard conditions approximately at 1 bar. *WLR*_std_ is also reported by OFM based on oil and water standard phase flow rates obtained using *PVT* relations. In all cases, *σ*_dh/std_ > 0.90, and for the majority of the data, *σ*_dh/std_ > 0.95.

**Figure 7:**
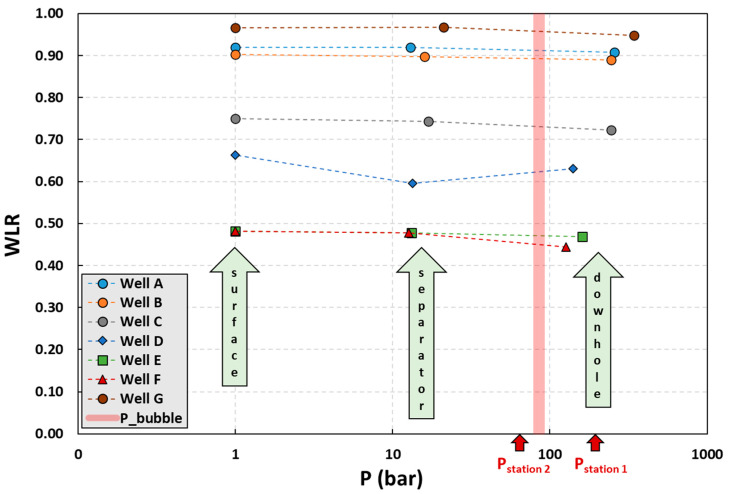
The slight increase in the water-in-liquid ratio (*WLR*) from downhole to standard conditions at the surface provides additional confidence that the *WLR* at Station 2 with a pressure just below the bubble-point pressure (*P*_b_) is expected to be very close to the downhole *WLR* at Station 1.

In its typical use of the current methodology, the pressure at Station 2 is not far from *P*_b_. Thus, it is expected that the ratio of *WLR*s at Stations 1 and 2 should be significantly larger than 0.95 (i.e., *σ*_1/2_ > 0.95). Note that the upper value of *σ* is bounded by 1.00, which represents the case of two-phase flow with no free gas in the mixture. Furthermore, for the special case where we have only one measurement station with the location pressure fluctuating around *P*_b_, the ratio *σ*_1/2_ is even closer to unity. This can further be visualized in the linear-log type semi-log graph in Figure 7 by observing the values of *WLR* at different measurement locations. The values of *WLR* at three measurement locations, namely, downhole, separator, and surface, are plotted for seven wells (Wells A through G). The downhole and surface *WLR* measurements are from the OFM and the 10+ bar measurements are from the test separator. The main observation is that as the fluid mixture moves from downhole to surface, the *WLR* values increase only slightly. The *P*_b_ band in Figure 7 represents the range of *P*_b_ values for the wells (i.e., 83–87 bar, as provided in Table 3). The downhole measurements represent the Station 1 measurements as indicated by *P*_station1_, while the Station 2 measurements are indicated by *P*_station2_. When the current methodology is implemented, Station 2 *WLR* measurements are expected to be very close to the Station 1 *WLR* measurements.

The slight difference demonstrated between the *WLR* values obtained at Stations 1 and 2 is not necessarily a requirement unless the methodology is implemented to obtain a first-order approximation of *WLR*_2_ without converting *WLR*_1_ from *P*_1_, *T*_1_ conditions to *P*_2_, *T*_2_ conditions. As explained earlier, in association with Figure 5, it is normally recommended (and in fact required if the stations are too far apart) that *WLR*_2_ should be adjusted for the *P*_2_, *T*_2_ conditions.

## 4. Summary and Conclusions

The current work introduces a new methodology to measure three-phase flow rates downhole. The methodology can be used with optical- or electronic-based measurement systems capable of measuring SoS and flow *V*. The concept is based on the measurements of SoS at different locations along the well where the pressure is greater than the bubble-point pressure at the first location and smaller at the second location. The fluid at the first location is a mixture of two phases, whereas at the second location, it becomes a mixture of three phases due to the liberation of gas from the oil phase. The flow equations are first solved for the two-phase flow at the first location to obtain the first phase fraction, *WLR*, and then this information is fed into the flow equations after adjustment to local *P* and *T* conditions to solve for the three-phase flow at the second location to obtain the second phase fraction, *LVF*. These two phase fractions, *WLR* and *LVF*, along with the bulk *V* at the second location are sufficient to calculate the three-phase flow rates.

The methodology may be implemented by using various sensor systems that could be based on different technologies. These sensors may be strategically positioned in the well to obtain the required measurements to solve the three-phase flow. It is also possible to use the same sensor technology effectively. For example, a DAS system that can provide SoS measurements on any section of the fiber-optic cable along the well would be an excellent choice for the implementation of this methodology. In this case, the two measurement locations may also be optimized to increase the accuracy of the measurement. Furthermore, this methodology may also be implemented for some special cases in which SoS is measured at the same location, but at different times. If the pressure at the measurement location is fluctuating around the bubble-point pressure, one measurement device capable of measuring SoS and *V* at that location may provide three-phase flow measurements by creating a feedback cycle between the two-phase and three-phase flow solution models.

Finally, the methodology introduced in this work can be retrofitted to existing wells with an optical infrastructure by adding an appropriate topside optoelectronics system and implementing and automating the methodology in the system’s flow solution algorithm.

## 5. Patents

This work is based on the patent application “Downhole 3-Phase Flow Measurement Using Speed of Sound Above and Below the Bubble-Point Pressure” (US Patent Application No. 17/660753).

## Figures and Tables

**Table 1:** Various downhole sensors and measurements that can be used in the calculation of three-phase flow rates.

Sensors	Image	Measurements
OFM ▪ Optical flowmeter (integrated with *P*/*T* gauge)	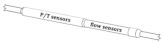	SoS, *V*, *P*, *T*
DAS_coil_ ▪ Distributed acoustic sensing (coil or helical configuration)	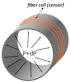	SoS, *V*
DAS_line_ ▪ Distributed acoustic sensing (straight-line configuration)	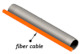	SoS
DTS and DPS ▪ Distributed temperature sensing system ▪ Distributed pressure sensing system	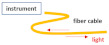	*T* *P*
Venturi ▪ Single-phase flowmeter: measures a differential pressure (Δ*P*) across two different cross-sectional areas ▪ Optical or electronic	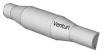	Δ*P* = *P*_1_ − *P*_2_
Δ*P* gauge ▪ Sensor which measures a Δ*P* across two points ▪ Optical or electronic	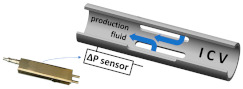	Δ*P* = *P*_1_ − *P*_2_
*P* gauge ▪ Pressure sensor ▪ Optical or electronic	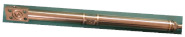	*P*
*T* gauge ▪ Temperature sensor ▪ Optical or electronic	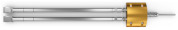	*T*

**Table 2:** Measurements by major sensor configurations at Stations 1 and 2 for the calculation of three-phase flow rates.

Configuration	Station 1 (*P* > *P*_b_)	Station 2 (*P* < *P*_b_)	Measurements
1	DAS_line_, *P*/*T*	DAS_coil_, *P*/*T*	SoS_1_, SoS_2_, *V*_2_, *P*_1_, *P*_2_, *T*_1_, *T*_2_
2	DAS_line_, DTS, DPS	DAS_coil_, DTS, DPS	SoS_1_, SoS_2_, *V*_2_, *P*_1_, *P*_2_, *T*_1_, *T*_2_
3	DAS_line_, *P*/*T*	OFM	SoS_1_, SoS_2_, *V*_2_, *P*_1_, *P*_2_, *T*_1_, *T*_2_
4	DAS_line_, *P*/*T*	DAS_line_, *P*/*T*, Venturi	SoS_1_, SoS_2_, *V*_2_, *P*_1_, *P*_2_, *T*_1_, *T*_2_
5	DAS_line_, *P*/*T*	DAS_line_, *P*/*T*, Δ*P* gauge	SoS_1_, SoS_2_, *V*_2_, *P*_1_, *P*_2_, *T*_1_, *T*_2_
6	OFM	OFM	SoS_1_, SoS_2_, *V*_1_, *V*_2_, *P*_1_, *P*_2_, *T*_1_, *T*_2_

**Table 3:** Generic descriptions of North Sea wells and corresponding pressures at the OFM locations.

Field	Well	Type	*P*_b_ (bar) (Bubble-Point)	*P* (bar) (at OFM)	*P* (bar) (Potential Low)	Equipment (Above OFM)	Equipment (Below OFM)
North Sea—Y	Y-1	platform producer	87	88–140	78	2 GLV, 1 CIV	ICV, ICD, SS, Packer
North Sea—Y	Y-2	platform producer	83	80–130	71	2 GLV, 1 CIV	ICV, ICD, SS, Packer
North Sea—Y	Y-3	platform producer	83	84–170	75	2 GLV, 1 CIV	ICV, ICD, SS, Packer

GLV: gas lift valve; CIV: chemical injection valve; ICV: inflow control valve; ICD: inflow control device; SS: sand screen.

## Data Availability

The data presented in this study are available on request from the corresponding author. The data are not publicly available due to third party restrictions.

## Appendix A. Prediction of Downhole Phase Fraction

### Appendix A.1. Definitions and Driving Equations

The relationship between the volumetric fractions of pure phase components in a multiphase flow mixture (i.e., oil, water, and gas) can be given by the following relation (Equation (A1)):

(A1) $$\phi_{o}+\phi_{w}+\phi_{g}=1$$

where *ϕ* represents the volumetric fraction and the indices “*o*, *w*, *g*” represent the oil, water, and gas phases, respectively.

Let us now define phase fractions in a given fluid mixture that will be used frequently: *LVF* represents the total liquid amount in a flowing fluid mixture (Equation (A2)) and *WLR* represents the amount of water in the total liquid flow (Equation (A3)):

(A2) $$LVF=\frac{\phi_{o}+\phi_{w}}{\phi_{o}+\phi_{w}+\phi_{g}}=\phi_{o}+\phi_{w}$$

(A3) $$WLR=\frac{\phi_{w}}{\phi_{o}+\phi_{w}}=\frac{\phi_{w}}{LVF}$$

Now, the volumetric fractions can be written in terms of phase fractions *LVF* and *WLR* (Equation (A4)):

(A4) $$\phi_{o}=\left(1-WLR\right)\left(LVF\right)\;\phi_{w}=\left(WLR\right)\left(LVF\right)\;\phi_{g}=\left(1-LVF\right)$$

The mixture density at the meter location downhole is given by the following relation:

(A5) $$\rho_{m}=\phi_{o}\rho_{o}+\phi_{w}\rho_{w}+\phi_{g}\rho_{g}$$

Substituting the phase fraction relations given by Equation (A4) into Equation (A5) results in the following:

(A6) $$\rho_{m}=\left(1-WLR\right)\left(LVF\right)\rho_{o}+(WLR)\left(LVF\right)\rho_{w}+\left(1-LVF\right)\rho_{g}$$

Equation (A6) is the mixture density relation written in terms of phase fractions *LVF* and *WLR*, and we frequently use this expression.

Fluid compressibility has the following relationship with density and speed of sound:

(A7) $$\kappa =\frac{1}{\rho a^{2}}$$

where *κ* denotes the fluid compressibility, *ρ* is the fluid density, and *a* is the SoS in the infinite fluid medium.

When dealing with multiphase flows, the typical approach is to use a volumetric proportion of each phase to calculate the mixture compressibility. In a three-phase well-mixed flow of oil/water/gas, the compressibility of the mixture, *κ*_m_, can be written as

(A8) $$\kappa_{m}=\phi_{o}\kappa_{o}+\phi_{w}\kappa_{w}+\phi_{g}\kappa_{g}$$

The mixture compressibility (Equation (A8)) can be written in the following form by applying Equation (A7) to Equation (A8):

(A9) $$\frac{1}{\rho_{m}a_{m}^{2}}=\frac{\phi_{o}}{\rho_{o}a_{o}^{2}}+\frac{\phi_{w}}{\rho_{w}a_{w}^{2}}+\frac{\phi_{g}}{\rho_{g}a_{g}^{2}}$$

The SoS values (*a_m_*, *a_o_*, *a_w_*, and *a_g_*) in this last expression (Equation (A9)) are for the infinite medium of the mixture, oil, water, and gas, respectively. Equation (A9) is sometimes called the Wood equation [25]. Now, we can rewrite Equation (A9) by using the phase fractions *LVF* and *WLR* given in Equation (A4):

(A10) $$\frac{1}{\rho_{m}a_{m}^{2}}=\frac{\left(LVF\right)\left(1-WLR\right)}{\rho_{o}a_{o}^{2}}+\frac{\left(LVF\right)\left(WLR\right)}{\rho_{w}a_{w}^{2}}+\frac{\left(1-LVF\right)}{\rho_{g}a_{g}^{2}}$$

For a confined area such as a pipe, the SoS measured by the flowmeter will be different as it will carry the compliance effects. The relationship between the SoS for the infinite medium and the SoS for the pipe is given by Equation (A11), the Korteweg-Lamb equation [26]:

(A11) $$\frac{1}{\rho_{m}a_{p}^{2}}=\frac{1}{\rho_{m}a_{m}^{2}}+\left(\frac{d}{t}\right)\left(\frac{1-\nu^{2}}{E}\right)$$

where

*a_p_*: SoS of mixture in the pipe (measured by sound measurement system);
*a_m_*: SoS of mixture in the infinite medium (used interchangeably with SoS_m_);
*d*: pipe diameter;
*t*: pipe wall thickness;
*E*: modulus of elasticity of pipe material;
*ν*: Poisson ratio of pipe material (0.3 for rigid bodies such as steel).

The Korteweg-Lamb equation can be rewritten by incorporating Wood equation (Equation (A10)):

(A12) $$\frac{1}{\rho_{m}a_{p}^{2}}=\frac{\left(LVF\right)\left(1-WLR\right)}{\rho_{o}a_{o}^{2}}+\frac{\left(LVF\right)\left(WLR\right)}{\rho_{w}a_{w}^{2}}+\frac{\left(1-LVF\right)}{\rho_{g}a_{g}^{2}}+\left(\frac{d}{t}\right)\left(\frac{1-\nu^{2}}{E}\right)$$

where *ρ_m_*, *ρ_o_*, *ρ_w_*, and *ρ_g_* refer to mixture, oil, water, and gas densities, respectively.

### Appendix A.2. Analytical Solutions for Phase Fractions

The two equations above (Equations (A6) and (A12)) can be rearranged to form the following general equation with the two unknowns, *WLR* and *LVF*.

(A13) $$\begin{array}{c} a_{p}^{2}\left[\left(\frac{1}{\rho_{w}a_{w}^{2}}-\frac{1}{\rho_{o}a_{o}^{2}}\right)WLR\cdot LVF+\left(\frac{1}{\rho_{o}a_{o}^{2}}-\frac{1}{\rho_{g}a_{g}^{2}}\right)LVF+\frac{1}{\rho_{g}a_{g}^{2}}+\left(\frac{d}{t}\right)\left(\frac{1-\nu^{2}}{E}\right)\right]\cdot \\ \left[\left({\rho_{w}-\rho }_{o}\right)WLR\cdot LVF+\left(\rho_{o}-\rho_{g}\right)LVF+\rho_{g}\right]-1=0 \end{array}$$

The current methodology uses Equation (A13) twice for different flow conditions. When *P* > *P*_b_, there is no free gas in the fluid mixture. Thus, *LVF* = 1, and the system is converted to a single equation with a single unknown.

(A14) $$a_{p}^{2}\left[\left(\frac{1}{\rho_{w}a_{w}^{2}}-\frac{1}{\rho_{o}a_{o}^{2}}\right)WLR+\frac{1}{\rho_{o}a_{o}^{2}}+\left(\frac{d}{t}\right)\left(\frac{1-\nu^{2}}{E}\right)\right]\cdot \left[\left({\rho_{w}-\rho }_{o}\right)WLR+\rho_{o}\right]-1=0$$

Rearranging for *WLR*, and solving for the quadratic equation,

(A15) $$\left[\underset{A}{\underbrace{a_{p}^{2}\left(\frac{1}{\rho_{w}a_{w}^{2}}-\frac{1}{\rho_{o}a_{o}^{2}}\right)}}WLR+\underset{B}{\underbrace{a_{p}^{2}\left\{\frac{1}{\rho_{o}a_{o}^{2}}+\left(\frac{d}{t}\right)\left(\frac{1-\nu^{2}}{E}\right)\right\}}}\right]\cdot \left[\underset{C}{\underbrace{\left(\rho_{w}-\rho_{o}\right)}}WLR+\underset{D}{\underbrace{\rho_{o}}}\right]-1=0$$

(A16) $$\left(A\cdot WLR+B\right)\cdot \left(C\cdot WLR+D\right)-1=0\;{WLR}^{2}+\underset{\beta }{\underbrace{\frac{\left(AD+BC\right)}{AC}}}\cdot WLR+\underset{\lambda }{\underbrace{\frac{BD-1}{AC}}}=0$$

(A17) $${WLR}_{1,2}=\frac{-\beta \pm \sqrt{\beta^{2}-4\lambda }}{2}$$

The roots of *WLR* are as follows:

(A18) $${WLR}_{1}=\left(-\beta +\sqrt{\delta }\right)/2\;\;{WLR}_{2}=\left(-\beta -\sqrt{\delta }\right)/2$$

where

$$\begin{array}{c} \beta =\frac{AD+BC}{AC}\\ \lambda =\frac{BD-1}{AC}\\ \delta =\beta^{2}-4\lambda \\ A=a_{p}^{2}\left(\frac{1}{\rho_{w}a_{w}^{2}}-\frac{1}{\rho_{o}a_{o}^{2}}\right)\\ B=a_{p}^{2}\left(\frac{1}{\rho_{o}a_{o}^{2}}+\left(\frac{d}{t}\right)\left(\frac{1-\nu^{2}}{E}\right)\right)\\ C=\left(\rho_{w}-\rho_{o}\right)\\ D=\rho_{o} \end{array}$$

Note that in a typical oil/water mixture, the valid root of Equation (A18) is always the positive root.

This *WLR* belongs to Station 1 at *P*_1_ and *T*_1_ conditions. It is recommended that *WLR* is adjusted for *P*_2_ and *T*_2_ conditions if the stations are too far apart. A first-order linear interpolation along the pressure interval between the two stations is explained in an earlier work [2,27]. When the *WLR* at Station 2 is known, Equation (A13) can be used to determine the *LVF* by measuring SoS_2_ at flow conditions when *P* < *P*_b_:

Rearranging Equation (A13) for the unknown *LVF*,
  (A19) $$\left[\underset{A^{*}}{\underbrace{a_{p}^{2}\left\{\left(\frac{1}{\rho_{w}a_{w}^{2}}-\frac{1}{\rho_{o}a_{o}^{2}}\right)WLR+\frac{1}{\rho_{o}a_{o}^{2}}-\frac{1}{\rho_{g}a_{g}^{2}}\right\}}}LVF+\underset{B^{*}}{\underbrace{a_{p}^{2}\left\{\frac{1}{\rho_{g}a_{g}^{2}}+\left(\frac{d}{t}\right)\left(\frac{1-\nu^{2}}{E}\right)\right\}}}\right]\cdot \;\left[\underset{C^{*}}{\underbrace{\left\{\left(\rho_{w}{-\rho }_{o}\right)WLR+\rho_{o}-\rho_{g}\right\}}}LVF+\underset{D^{*}}{\underbrace{\rho_{g}}}\right]-1=0$$
  (A20) $$\begin{array}{c} \left(A^{*}\cdot LVF+B^{*}\right)\left(C^{*}\cdot LVF+D^{*}\right)-1=0\\ {LVF}^{2}+\underset{\beta^{*}}{\underbrace{\frac{\left(A^{*}D^{*}+B^{*}C^{*}\right)}{A^{*}C^{*}}}}\cdot LVF+\underset{\lambda^{*}}{\underbrace{\frac{B^{*}D^{*}-1}{A^{*}C^{*}}}}=0 \end{array}$$
  (A21) $${LVF}_{1,2}=\frac{-\beta^{*}\pm \sqrt{\beta^{*2}-4\lambda^{*}}}{2}$$
  The roots of the *LVF* are as follows:
  (A22) $${LVF}_{1}=\left(-\beta^{*}+\sqrt{\delta^{*}}\right)/2\;\;{LVF}_{2}=\left(-\beta^{*}-\sqrt{\delta^{*}}\right)/2$$
  where
  $$\begin{array}{c} \beta^{*}=\frac{A^{*}D^{*}+B^{*}C^{*}}{A^{*}C^{*}}\\ \lambda^{*}=\frac{B^{*}D^{*}-1}{A^{*}C^{*}}\\ \delta^{*}=\beta^{*2}-4\lambda^{*}\\ A^{*}=a_{p}^{2}\left\{\left(\frac{1}{\rho_{w}a_{w}^{2}}-\frac{1}{\rho_{o}a_{o}^{2}}\right)WLR+\frac{1}{\rho_{o}a_{o}^{2}}-\frac{1}{\rho_{g}a_{g}^{2}}\right\}\\ B^{*}=a_{p}^{2}\left\{\frac{1}{\rho_{g}a_{g}^{2}}+\left(\frac{d}{t}\right)\left(\frac{1-\nu^{2}}{E}\right)\right\}\\ C^{*}=\left\{\left(\rho_{w}{-\rho }_{o}\right)WLR+\rho_{o}-\rho_{g}\right\}\\ D^{*}=\rho_{g} \end{array}$$

Unlike the *WLR* in the liquid/liquid solution, both roots of *LVF* are valid; the positive root represents a liquid-rich solution, whereas the negative root represents the gas-rich solution (Figure 14 of [19]). This is because the curve based on the Wood equation takes a minimum value within the possible *LVF* range (0 to 1) and thus for some SoS values a dual solution corresponding to positive and negative roots of *LVF* exist.

### Appendix A.3. Phase Flow Rates

The calculation of three-phase flow rates makes use of the available information provided in the parameter file, which includes tables of SoS and tables of other fluid properties such as density and viscosity of the individual phases.

Once the inline phase fractions *LVF* and *WLR* are determined, the inline phase flow rates can be calculated:

(A23) $$Q_{total}=V\cdot Area\;Q_{oil}=Q_{total}\cdot LVF\cdot \left(1-WLR\right)\;Q_{gas}=Q_{total}\cdot \left(1-LVF\right)\;Q_{water}=Q_{total}\cdot LVF\cdot WLR$$

where *Q* is the volumetric flow rate, *V* is the fluid velocity, and Area is the cross-sectional area of the pipe where the *V* measurement is made.

The corresponding inline mass flow rates can also be calculated:

(A24) $$M_{total}=\rho_{m}\cdot Q_{total}\;M_{oil}=\rho_{o}\cdot Q_{oil}\;M_{gas}={\rho_{g}\cdot Q}_{gas}\;M_{water}=\rho_{w}\cdot Q_{water}$$

where *M* is the mass flow rate.

The standard phase flow rates can be obtained using the inline phase flow rates and the conversion factors (derived from the *PVT* analysis of the initial fluid report) in the flowmeter’s parameter file.

The flow rate derivations using Equations (A23) and (A24) assume well-mixed flows. For the gas/liquid flows, it is also possible to implement various multiphase flow correlations to consider possible slip conditions between the phases. As an example, three different methods are mentioned here: (1) Homogeneous flow approach in which flow patterns and slippage between the phases are not considered (Equation (A23) is based on this approach); (2) an empirical correlation that predicts the liquid holdup and pressure gradient, while considering the slippage but not the flow pattern [33]; and (3) another empirical correlation that considers both slippage and flow patterns [31].
